# Efficacy of Combined XingZhi-YiNao Granules and Hyperbaric Oxygen Therapy for Cognition and Motor Dysfunction in Patients with Delayed Encephalopathy after Acute Carbon Monoxide Poisoning

**DOI:** 10.1155/2017/1323297

**Published:** 2017-11-26

**Authors:** Li Qin, Chu Meihua, Guo Dadong, Wang Li, Wang Jinglin, Ding Xiaoyu, Bi Mingjun, Zou Yong

**Affiliations:** ^1^Department of Integration of Chinese and Western Medicine, The Affiliated Yantai Yuhuangding Hospital of Qingdao University, Yantai, Shandong 264000, China; ^2^Eye Institute of Shandong University of Traditional Chinese Medicine, Jinan, Shandong 250002, China; ^3^Emergency Center, The Affiliated Yantai Yuhuangding Hospital of Qingdao University, Yantai, Shandong 264000, China; ^4^Department of Clinical Medicine, Qingdao University Medical College, Qingdao, Shandong 266003, China

## Abstract

**Purpose:**

To investigate the efficacy of XingZhi-YiNao (XZYN) granules and hyperbaric oxygenation (HBO) for cognition and motor dysfunction in patients with delayed encephalopathy after acute carbon monoxide poisoning (DEACMP).

**Methods:**

Eighty-nine patients with DEACMP were randomly divided into control group (*n* = 19), HBO group (*n* = 32), and XZYN group (*n* = 38). All patients received conventional treatment. HBO group received HBO therapy once daily. XZYN group received extra XZYN granules plus HBO treatment. The related indexes including activity of daily living (ADL) scale, Montreal cognitive assessment (MoCA) scale, and mini mental state examination (MMSE) scale were measured. Cerebral white matter injury, age related white matter changes (ARWMC) scale, and the amplitude and latency of P300 were assessed.

**Results:**

Compared with control group, the neurological function scores of ADL, MoCA, and MMSE in HBO and XZYN groups were significantly improved, the impairment degree of brain white matter and cognition function were obviously alleviated, the latencies of P300 were significantly shortened, and the amplitudes of P300 were evidently increased (*P* < 0.05). Treatment efficacy of XZYN group was superior to that of HBO group (*P* < 0.05).

**Conclusion:**

Combined XZYN granules and HBO can significantly improve cognition and motor functions in patients with DEACMP.

## 1. Introduction

Nowadays, carbon monoxide (CO) poisoning is the most common health problem for people in many countries [1]. It can lead to a lot of neurophysiology and neuropathological changes, among which acute brain injury and delayed encephalopathy after carbon monoxide poisoning (DEACMP) are the most common neurological complications [2]. Multiple factors have been found to be associated with the development of DEACMP, such as ischemia and hypoxia, cytotoxic injury, reperfusion injury, immune dysfunction, and neurotransmitter dysregulation, resulting in overlapping processes [3, 4]. Nevertheless, the specific mechanisms of DEACMP are still unclear. Currently, mounting evidence suggests hyperbaric oxygen (HBO) therapy as the best treatment for patients with acute CO poisoning, but its effectiveness for the prevention and treatment in DEACMP remains controversial [5, 6] because approximately 10% to 30% patients finally develop DEACMP despite successful HBO treatment of the initial symptoms during one session or repetitive sessions. In terms of the medicine application, more and more studies have paid attention to corticosteroids [7], antioxidants, and free radical scavengers [8, 9]. Other drugs, such as brain-protective agents, traditional Chinese medicine (TCM), and natural animal and plant extracts [10–12], have also been applied to clinical practice. However, these drugs can not fundamentally prevent or inhibit the occurrence and development of either acute brain damage or DEACMP due to the lack of evidence-based medicine. It has been demonstrated that the early application of TCM can improve brain microcirculation, promote nerve cell survival, and benefit neurological recovery [13]. Therefore, TCM may play a role in repairing the structure and function of damaged nerve cells, improving the neurological function in patients with severe acute CO poisoning and DEACMP.

Based on the manifestation of patients with DEACMP, we fabricated the formulation of “XingZhi-YiNao (XZYN) granules” using a therapeutic method of “detoxifying, promoting blood circulation, dissolving phlegm, removing obstruction, unraveling wisdom, and replenishing essence.” The XZYN granules include 8 types of boil-free granules of traditional Chinese medicine extracts as follows: Coptis Rhizome, 10 g (Batch Number: 1701081, Anhui Puren Chinese Medicine Slices Co., Ltd.); Panax Ginseng, 10 g (Batch Number: 160501, Hubei Didao Medicinal Materials Technology Co., Ltd.); Poria cum Radix Pini, 10 g (Batch Number: 161111, Anhui Puren Chinese Medicine Slices Co., Ltd.);* Acorus tatarinowii* Schott, 20 g (Batch Number: 1707092, Anhui Puren Chinese Medicine Slices Co., Ltd.); Milkworts, 10 g (Batch Number: 1704262, Anhui Puren Chinese Medicine Slices Co., Ltd.);* Salvia miltiorrhiza*, 30 g (Batch Number: 1707103, Anhui Puren Chinese Medicine Slices Co., Ltd.); Radix Puerariae, 10 g (Batch Number: 1703042, Anhui Puren Chinese Medicine Slices Co., Ltd.); Bile Arisaema, 10 g (Batch Number: 160503, Sichuan Fuzheng Pharmaceutical Co., Ltd.). All boil-free granules were purchased from Shandong green leaf Medical & Pharmaceutical Co., Ltd. (Shandong, China). The identification for each granule was determined by thin layer chromatography (Qingdao Haiyang Chemical Co. Ltd., Qingdao, China), and the quality of all products met the requirements of Chinese Pharmacopeia [14]. In this study, we aimed to explore the efficacy of TCM in patients with DEACMP through retrospective case series analysis, offering a foundation for the clinical application of TCM in CO poisoning.

## 2. Material and Methods

### 2.1. Recruited and Excluded Criteria

Patients diagnosed with DEACMP were recruited in the Affiliated Yantai Yuhuangding Hospital of Qingdao University from June 2011 to May 2016. The criteria for DEACMP were as follows: (1) history of CO exposure; (2) age from 18 to 75 years; (3) clinical symptoms of DEACMP occurring after 2~60 days of “latent period” without any clinical manifestation of acute CO poisoning; (4) demyelination lesions of bilateral cerebral white matter in brain MRI; (5) patients and their family members signing informed consent and completing all of the treatment program and a follow-up plan. In this study, those who had chronic hepatic and renal insufficiency, diabetes, coronary heart disease, cerebrovascular disease, malignant tumor, drug poisoning or addiction, radioactive encephalopathy, encephalitis, multiple sclerosis, sarcoidosis, mild cognitive impairment, or dementia diagnosed before the poisoning onset or midway death or failure to complete follow-up planning were excluded from the final statistics. Follow-up examinations were performed from 2011 to 2017.

### 2.2. Participants and Treatment Strategy

A total of 112 patients with DEACMP were recruited at first as the primary endpoint. Before treatment, 23 cases were excluded because they did not agree to randomization or failed to complete the follow-up. Finally, 89 patients were selected to participate in the study and then randomly divided into a control group (*n* = 19), a hyperbaric oxygen treatment group (HBO group, *n* = 32), and a XingZhi-YiNao granules treatment group (XZYN group, *n* = 38). For every patient, the detailed information associated with the demographic and clinical parameters, such as sex, age, body mass index (BMI), education level (years), work type, CO exposure time, latent phase, coma time, blood carboxyhemoglobin level, S100B protein level, and lactate clearance rate (%), was listed in Table 1. No statistical differences were found among the three groups before treatment (*P* > 0.05). Meanwhile, all patients received nasal tube of oxygen inhalation and conventional treatment with regulating water and electrolytes, maintaining stable blood pressure and blood glucose, and injecting cytidine diphosphate choline (CDPC) and vitamin B. Patients in HBO group underwent extra hyperbaric oxygen therapy once daily till 2 months, while the cases in XZYN group received XingZhi-YiNao granules twice daily based on the treatment options of HBO group. Clinical manifestations of the patients were recorded by two trained neurologists, and laboratory studies, including blood routine, blood lipid, blood glucose, electrolytes and liver and kidney function, brain MRI, and neural electrophysiology, were performed before and within 72 hours after treatment in order to understand the unpleasant side effects induced by drugs.

### 2.3. Experimental Methods

#### 2.3.1. Neurological Function Assessment

The movement and cognition changes of patients were measured by activities of daily living (ADL) scale, Montreal cognitive assessment (MoCA) scale, and mini mental state examination (MMSE) before and after treatment at 1 month and 2 months, respectively. The total ADL score was set to 100, and the higher the score, the better the ability of daily life of patients. In the meantime, the total MMSE and MoCA scores were both set to 30, and the higher score indicated the stronger cognitive ability of patients. However, a low score (<26) was regarded as cognitive dysfunction. The neurological function assessment of all patients was evaluated by two trained raters in a double-blind manner at the same time point. We described our continuous data by their mean value and standard deviation or median (interquartile range) in the final statistics.

#### 2.3.2. Neuroimaging and White Matter Lesions Score

All patients underwent routine MRI scanning of brain on a Philips Intera system (Philips Medical Systems, Best, the Netherlands). At 1.5 T, an axial 2D spin echo sequence with the following sequence parameters was acquired: repetition time/echo time/inversion time (TI)/flip angle (FA) = 540 ms/14 ms/ms/90°, matrix = 256 × 256, 22 slices, thickness = 5 mm, and spacing at 6 mm. The white matter lesions were evaluated by the age related white matter change (ARWMC) scale [15] and the revised scale of Xiong et al. [16] as described in Table 2. The total scale score was 30. The higher the score, the more serious the white matter lesions and the wider the damage scope. All scores were performed by two physicians simultaneously in a double-blind manner, and the average value was calculated and expressed in the final statistics.

#### 2.3.3. P300 Detection

The latency and amplitude of P300 were recorded using a electromyography evoked potentiometer of Japanese Photoelectric MEB-9200K. All tests were performed in a double-blind manner by two experienced professionals.

### 2.4. Statistical Analysis

All statistical analyses were performed using SPSS17.0 statistical software, and all the continuous variables were expressed as mean ± standard deviation. Analysis of covariance (ANCOVA) was conducted to observe the difference effect of treatments on neurological function scores among three groups. Student's test and the chi-squared test were applied to investigate the differences between the two groups on demographic and baseline clinical variables. All procedures were 2-tailed and a value of *P* < 0.05 was considered statistical for all tests.

## 3. Results

### 3.1. Comparisons of ADL, MoCA, MMSE, and ARWMC Scores among Three Groups

Based on the statistical results, we found that there was no significant difference in ADL, MoCA, and MMSE scores among three groups prior to treatment (*P* > 0.05). However, the scores of ADL, MoCA, and MMSE in both HBO group and XZYN group were significantly higher than those in control group after treatment (*P* < 0.05). Moreover, compared with those in HBO group, the scores of ADL, MoCA, and MMSE in XZYN group were apparently elevated at the same time point (Table 3, *P* < 0.05). This result suggested that hyperbaric oxygen can improve the daily exercise ability and cognitive function of patients with DEACMP, and the efficacy of combined HBO and XZYN granules is more effective in patients with DEACMP.

Imaging data showed that many abnormal performances could be identified in patients with DEACMP to some extent, mainly as follows: long T1, long T2 signals in MRI, slightly higher signal in FLAIR, and high signal in DWI, with low signal in diffusion coefficient (ADC). Brain lesion often lies in cerebral cortex of occipital lobe, semioval center, white matter around lateral ventricle, and globus pallidus and could be observed in white matter and deep gray matter nucleus. Some coexistence or fusion of multiple lesions could also be showed in part of subjects with DEACMP. There was no significant difference in ARWMC score among three groups before treatment (*P* > 0.05). The ARWMC score was slightly decreased at 1 month after intervention treatment in both HBO and XZYN groups, but no statistical difference was found as compared with NC group (*P* > 0.05). The score of ARWMC was significantly lower in both HBO and XZYN groups at 2 months than in control group (Table 3, *P* < 0.05), suggesting that hyperbaric oxygen therapy can significantly reduce the lesion degree of white matter in patients after CO poisoning, and the combined application of TCM and hyperbaric oxygen therapy may be more conducive to neurological recovery in patients with DEACMP.

### 3.2. Comparison of Event-Related Potential P300 among Three Groups before and after Treatment

Event-related potentials (P300) is an objective index to reflect the severity and the prognosis judgment of patients with cognitive impairment [17, 18]. In the present study, we noted that, before treatment, there were no significant differences in the latency and amplitude of P300 among three groups (*P* > 0.05). However, the latency of P300 in patients was significantly shortened and the amplitude was significantly higher after treatment with combined hyperbaric oxygen and XZYN granules, especially at 1 month, accompanied by a statistical difference compared with the control group (Table 4, *P* < 0.05). We also found that the P300 latency in XZYN group was significantly shorter and the amplitude was higher than that of HBO group at the same time point (*P* < 0.05), suggesting that hyperbaric oxygen therapy combined with XZYN granules can significantly improve the cognitive function of patients with delayed encephalopathy after CO poisoning.

### 3.3. Adverse Events

No apparent adverse reactions of the drugs were found in the included patients. There were no significant alterations for blood glucose, blood lipids, and liver and kidney function during the whole treatment session.

## 4. Discussion

DEACMP is the most common neurological complication after acute CO poisoning. The main manifestations are dyskinesia, advanced intelligence loss, and mental behavior abnormality, which seriously affect the life quality of the patients. However, the detailed pathogenesis of DEACMP is unclear. Most scholars believed that ischemia and hypoxia, neuronal apoptosis and necrosis, the increased excitatory amino acid, neurotransmitter abnormalities, oxidative stress, and lipid peroxidation products caused by CO poisoning could lead to the structural and functional changes of myelin basic proteins, the abnormal expression of myelin-derived axonal growth inhibitors (such as Nogo, NgR, and Ogmp), and the imbalanced-expression of cytokines and inflammatory molecules in the central nervous system, which may be an important factor of generalized demyelination lesions in white matter of brain tissue [19, 20]. ADL, MoCA, and MMSE are the most common indicators to evaluate the functions of movement and cognition of patients. ARWMC is mainly used for the quantitative evaluation of white matter lesions and easy to operate with good credibility. Its characteristic is that it can rapidly and quantitatively judge the size and severity of the lesions in white matter and basal ganglia area. Our results showed that the scores of ADL, MoCA, and MMSE in patients with DEACMP were significantly lower than the average, while the extensive demyelinating lesions were found in brain tissue in all three groups before treatment, and the score of ARWMC was obviously increased, suggesting that the patients had obvious motor and cognitive dysfunctions. However, after the comprehensive treatment of combined hyperbaric oxygen and XZYN granules, the motor and cognitive functions of patients were improved significantly; some demyelinating lesions were recovered. This evidently indicated the reversibility of motor and cognitive disabilities and white matter lesions in DEACMP patients to a certain extent [2, 21]. The treatment of combined hyperbaric oxygen and XZYN granules achieved better efficacy in DEACMP patients in our study, and it is worth further clinical use.

Event-related potential P300 is an important indicator for brain cognitive and psychological state, and participants were required to engage in cognitive activities, including attention, alertness, working memory, executive function, and many other cognitive functions. Currently, the determination of P300 has been widely used in clinical practice. However, the amplitude of P300 is also affected by a variety of stimulus parameters, such as the probability, the order and the quality of stimulation, the difficulty of the task, the sensitivity of the reward, and the emotional input of the subjects. The increased amplitude of P300 may reflect an urgent adaptation and regulatory processing. In the present study, we found that the latency of P300 was prolonged and the amplitude was decreased in patients with DEACMP to some extent. Statistical analysis revealed that the latency of P300 was related to the scores of MoCA and MMSE in DEACMP patients, suggesting that P300 latency could be used as an important index to judge the cognitive function of patients. The latency of P300 is associated with MoCA and MMSE scores in DEACMP patients, suggesting that P300 latency can be used as an important index to judge the cognitive function of patients. However, the relationship between P300 latency and the scores of ADL and ARWMC is uncertain in our study. We suspect that it is mainly concerned with the following aspects: (1) P300 is a complex waveform, which can objectively reflect the nerve fiber contact in cerebral cortex and cognitive activity participation in different brain areas, and is closely related to memory, cognitive, calculation, understanding, judgment, and reasoning functions. P300 latency reflects the speed of neural activity and processing, relating to the intensity of attention, memory, and cognitive function. The ADL score mainly evaluates the daily life ability of the patients, and the state of consciousness, the mental state, the cognitive function, and the limb movement condition of the patients can result in the decrease of ADL score. ARWMC is currently used to assess the severity and extent of white matter lesions. Based on the aforementioned results, we found that white matter lesions in DEACMP patients were common in the cortex of parietal and occipital lobe, semioval center, white matter around lateral ventricle, and globus pallidus, and there were different degrees of damage in the white matter and deep gray nuclei. When nerve fiber demyelination and other ARWMC typical changes occur in these brain areas, these will slow or block information transmission of cortical neurons, which ultimately led to signal disruption of different brain functional areas, resulting in cognitive dysfunction. However, individuals with DEACMP exhibited comprehensive cognitive dysfunction, and the clinical manifestations slightly varied during each session of the disease. The memory, language, and the visual function of DEACMP patients were rapidly declined in a very short term, and personality abnormalities and other cognitive functions (including generalization, judgment, and multiability in analyzing and solving questions) appeared at a later stage and seriously and often accompanied with emotional disorders and extrapyramidal and pyramidal system dysfunction, which may directly affect the cognitive activities of the participants involved in the study. This can explain why P300 is related to the scores of ADL and ARWMC. (2) All patients were enrolled voluntarily and were required to complete all the trials. And thus, those who failed to cooperate with the research or lost following up or had suffered either serious illness or half-way death were excluded from the statistics. Thus, a relatively smaller sample size, younger population, and milder poisoning were included as compared with the general DEACMP patients and thus may affect the accuracy of the study.

## 5. Conclusions

In summary, this study revealed that the combined application of traditional Chinese medicine XingZhi-YiNao granules and HBO could significantly improve cognitive function and motor function in patients with delayed encephalopathy after acute carbon monoxide. However, it needs to verify and support the efficacy of this method from more patients and further research in the future.

## Figures and Tables

**Table 1:** Baseline characteristics and clinical data of the included patients.

Variable	Control group	HBO group	XZYN group	*q*/*χ*^2^	*P* value
Number	19	32	38		
Female/male	10/9	18/14	21/17	0.0062	*P* > 0.05
Age (y)	47.2 ± 7.5	48.5 ± 6.8	47.6 ± 8.2	1.213~1.835	*P* > 0.05
BMI (kg/m^2^)	22.3 ± 3.5	21.7 ± 3.2	22.5 ± 3.7	0.758~1.136	*P* > 0.05
Education level (y)	10.1 ± 4.2	9.8 ± 4.4	9.2 ± 4.9	0.892~2.015	*P* > 0.05
Work type (Me/Ma)	12/7	22/10	25/13	0.1869	*P* > 0.05
CO exposure time (h)	9.6 ± 7.2	10.8 ± 6.8	10.1 ± 7.5	1.270~1.753	*P* > 0.05
COHb levels (%)	28.0 ± 12.5	25.7 ± 11.2	26.9 ± 13.2	1.305~2.281	*P* > 0.05
Latent phase (d)	15.2 ± 7.6	13.5 ± 7.4	15.8 ± 8.1	1.208~1.913	*P* > 0.05
Coma time (h)	6.8 ± 3.6	7.0 ± 3.2	6.5 ± 3.9	1.422~2.148	*P* > 0.05
Serum S100B (*μ*/L)	0.8 ± 0.3	0.7 ± 0.2	0.8 ± 0.5	1.270~1.695	*P* > 0.05
Lactate clearance rate (%)	39.3 ± 6.2	38.7 ± 7.1	37.9 ± 6.4	1.369~2.512	*P* > 0.05

BMI: body mass index; Me: mental worker; Ma: manual worker; COHb: carboxyhemoglobin.

**Table 2:** Operational definitions of ARWMC scale.

Score	Frontal lobe, parietal-occipital lobe, temporal lobe, and infratentorial region	Basal ganglia
0	No lesions (PVWMC: symmetrical, well-defined ventricle, or DWMC < 5 mm)	No lesions
1	Focal lesions (PVWMC, nonsymmetrical caps or bands < 5 mm, or DWMC between 5 and 9 mm)	One focal lesion (>5 mm)
2	Beginning confluence of lesions (PVWMC between 5 and 10 mm or DWMC between 10 and 25 mm or connecting bridge between two focal lesions)	More than one focal lesion
3	Diffuse involvement of most region (PVWMC > 10 mm or DWMC > 25 mm)	Confluence of lesions

ARWMC, age related white matter changes; PVWMC, periventricular white matter changes; DWMC, deep white matter changes. This table is from [16].

**Table 3:** Comparison of ADL, MoCA, MMSE, and ARWMC at different time points before and after treatment in each group.

Group	*n*	ADL	MoCA	MMSE	ARWMC
Control group					
Before treatment	19	12.4 ± 5.8	15.1 ± 3.5	14.1 ± 4.3	18.7 ± 4.1
1 month after treatment	19	21.5 ± 6.6	16.7 ± 4.0	15.9 ± 3.0	17.5 ± 3.2
2 months after treatment	19	23.0 ± 6.1	18.2 ± 3.6	18.1 ± 4.5	15.2 ± 3.3
HBO group					
Before treatment	24	13.8 ± 6.0	14.6 ± 3.9	13.7 ± 4.6	19.0 ± 3.3
1 month after treatment	24	46.9 ± 7.7^a^	20.5 ± 3.0^a^	18.0 ± 3.2^a^	16.1 ± 3.1
2 months after treatment	24	60.5 ± 8.1^a^	22.2 ± 2.7^a^	22.4 ± 3.5^a^	8.7 ± 2.2^a^
XZYN group					
Before treatment	24	12.9 ± 5.1	15.5 ± 4.2	14.4 ± 3.8	18.1 ± 3.9
1 month after treatment	24	57.6 ± 6.4^ab^	23.3 ± 2.9^ab^	21.4 ± 3.5^ab^	13.2 ± 3.3^ab^
2 months after treatment	24	70.2 ± 8.3^ab^	26.1 ± 3.1^ab^	25.9 ± 4.1^ab^	7.0 ± 2.1^ab^

*Note*. Compared with the control group at the same time point, ^a^*P* < 0.05; compared with HBO group at the same time point, ^b^*P* < 0.05.

**Table 4:** Comparison of event-related potential P300 among three groups.

Group	*n*	Latency of P300			Amplitude of P300		
		Before treatment	1 month after treatment	2 months after treatment	Before treatment	1 month after treatment	2 months after treatment
Control group	19	410.3 ± 32.5	390.2 ± 27.5	381.7 ± 30.3	3.8 ± 1.1	4.0 ± 1.0	4.1 ± 1.5
HBO group	24	407.6 ± 30.9	373.6 ± 28.7^a^	352.5 ± 23.6^a^	3.7 ± 1.6	4.5 ± 0.9^a^	5.6 ± 1.3^a^
XZYN group	24	412.1 ± 34.0	370.4 ± 25.3^a^	332.9 ± 20.4^ab^	3.7 ± 1.3	5.3 ± 1.2^ab^	6.5 ± 1.6^ab^

*Note*. Compared with the control group at the same time point, ^a^*P*< 0.05; compared with HBO group at the same time point, ^b^*P* < 0.05.

## Abbreviations

ADL: Activity of daily living
ARWMC: Age related white matter changes
CO: Carbon monoxide
COHb: Carboxyhemoglobin
DEACMP: Delayed encephalopathy after acute carbon monoxide poisoning
HBO: Hyperbaric oxygenation
MMSE: Mini mental state examination
MoCA: Montreal cognitive assessment
TCM: Traditional Chinese medicine.
